# Electroconvulsive therapy for an older patient with schizophrenia complicated by nonconvulsive status epilepticus during catatonia: A case report

**DOI:** 10.1002/pcn5.70138

**Published:** 2025-06-16

**Authors:** Ayumi Takeshita, Masaya Mashimoto, Hiromi Chiba, Motohiro Ozone

**Affiliations:** ^1^ Department of Neuropsychiatry Kurume University School of Medicine Kurume‐city Fukuoka Japan

**Keywords:** catatonia, electroconvulsive therapy, nonconvulsive status epilepticus, schizophrenia, stupor

## Abstract

**Background:**

Nonconvulsive status epilepticus (NCSE) can present with symptoms resembling catatonia, such as stupor, staring, and immobility. Distinguishing between the two conditions using electroencephalography (EEG) is crucial. However, reports of NCSE coexisting with catatonia are rare.

**Case Presentation:**

We present a case of catatonia associated with schizophrenia complicated by NCSE. A 77‐year‐old woman with a 30‐year history of well‐controlled schizophrenia developed stupor and was admitted to our hospital. EEG revealed evolving spike‐and‐wave complexes, leading to a diagnosis of NCSE. Administration of levetiracetam improved the EEG findings, and subsequent monitoring confirmed resolution of epileptiform activity. However, the patient's stuporous state persisted despite the normalized EEG. Extensive workup showed no evidence of encephalitis or other neurological pathology. We diagnosed her with NCSE and catatonia associated with schizophrenia. Electroconvulsive therapy (ECT) was administered, resulting in complete resolution of the catatonic symptoms.

**Conclusion:**

This case highlights three key points. First, stupor can result from both NCSE and catatonia associated with schizophrenia. Second, when no physical cause for NCSE is identified and symptoms persist despite EEG improvement following antiepileptic treatment, coexisting catatonia associated with schizophrenia should be considered. Finally, ECT was effective in treating catatonia associated with schizophrenia complicated by NCSE. In patients presenting with stupor, it is important to differentiate between NCSE and catatonia associated with schizophrenia and to recognize the potential for their coexistence.

## BACKGROUND

Catatonia presents with a range of symptoms and has multiple underlying causes.^1^ According to the *Diagnostic and Statistical Manual of Mental Disorders, Fifth Edition* (DSM‐5),^2^ symptoms of catatonia include stupor, catalepsy, waxy flexibility, mutism, posturing, tics, stereotyped movements, agitation, facial grimacing, echolalia, and echopraxia.^3^ Catatonia is not only associated with psychiatric conditions, such as schizophrenia and depression, but can also occur in conditions like anti‐*N*‐methyl‐d‐aspartate (NMDA) receptor encephalitis, metabolic disturbances, focal cerebral lesions, and withdrawal from substances such as benzodiazepines, alcohol, and opioids.^3^ Furthermore, the association between catatonia and epilepsy has gained increasing attention.^4^ Nonconvulsive status epilepticus (NCSE), in particular, has been reported as a potential cause of catatonia.^5^

NCSE refers to status epilepticus without prominent motor activity.^6^ It can present with symptoms similar to those of catatonia, such as stupor, staring, and unresponsiveness, and is diagnosed based on electroencephalography (EEG) findings in conjunction with clinical symptoms.^5^ Diurnal variations in symptoms may occur, with manifestations appearing only during periods when EEG abnormalities are present.^5^ The causes of NCSE include epilepsy, brain lesions or damage, infections, drug effects, toxic ingestion, alcohol use, metabolic imbalances, and autoimmune diseases, such as systemic lupus erythematosus.^7^ Treatment for NCSE typically involves the use of antiseizure medications and management of the underlying cause.^8^

Here, we present a case of catatonic stupor in a patient with schizophrenia, complicated by NCSE. Given the overlap in clinical symptoms between catatonia associated with schizophrenia and NCSE, accurate differential diagnosis is crucial. However, to our knowledge, the only previously reported case of their coexistence is that by Suzuki et al.^9^ Informed consent for publication was obtained from the patient.

## CASE PRESENTATION

A 77‐year‐old woman presented with stupor and was admitted to A Hospital in Y‐1 month, X year. She had no family history of psychiatric disorders but had a medical history of hypertension. Her birth and developmental history were unremarkable, and she began working after graduating from junior high school. According to her son, the patient first developed catatonic symptoms at the age of 30; however, no medical records were available to substantiate this history, and the clinical details remain uncertain. She was subsequently diagnosed with schizophrenia, and her symptoms were reportedly well managed with pharmacological treatment.

In Y‐1 month, X year, she became confused, claiming that “someone is in the house.” A passing stranger called the police, and she was taken into protective custody before being admitted to A Hospital. On the 5th day of hospitalization, she remained bedridden and displayed minimal speech and only slight nodding. These findings were interpreted as indicative of stupor.

After starting lorazepam at 1.5 mg, she was able to consume small amounts of food with assistance. However, even after the dose was increased to 3 mg, her speech remained minimal. While capable of conversing slowly and deliberately, she refused oral intake, and intravenous fluid therapy continued. On the 10th day of hospitalization, she developed a fever, and aspiration pneumonia was suspected. Antibiotic therapy with ampicillin/sulbactam (6 g/day) was initiated, leading to prompt defervescence and restoration of her ability to talk. However, on the 14th day, the patient developed a high fever of 40°C and became stuporous again. The antibiotics were switched to tazobactam/piperacillin (13.5 g/day). A CT scan showed no evidence of pneumonia or other obvious sources of fever. Malignant catatonia was suspected due to autonomic symptoms, such as tachycardia, blood pressure fluctuations, and fever, along with stupor. She was subsequently transferred to our hospital in X year, Y month. Prior to transfer, her medications included nitrazepam 10 mg, olanzapine 5 mg, lorazepam 3 mg, and lemborexant 5 mg.

Upon admission to our hospital, her temperature was 37.9°C, with a heart rate of 110 bpm, blood pressure of 132/82 mmHg, and SpO₂ of 98% on room air. Her level of consciousness was assessed as GCS 6 (E4 V1 M1). While lying supine, her eyes remained open without blinking, and she showed no speech or response to verbal or painful stimuli. Bilateral pupillary light reflexes were intact, and no abnormalities in eye movement were noted. The neck was supple. Muscle tone in the extremities was increased, passive movement elicited resistance, and the patient maintaining a flexed posture. This posture was sustained when the upper and lower limbs were passively raised. Her total score on the Bush–Francis Catatonia Rating Scale was 27.

On the first day of hospitalization, due to her stuporous state and inability to take oral medications, a nasogastric tube was inserted. Lorazepam 3 mg (1 mg three times a day after meals) and lemborexant 5 mg were resumed. Electroconvulsive therapy (ECT) was planned, and nitrazepam 5 mg and olanzapine were discontinued. Laboratory tests upon admission revealed mild leukocytosis and elevated creatine kinase levels, while C‐reactive protein was negative (Table 1).

**Table 1:** Laboratory test results upon admission.

Laboratory test	Results	Normal range
White blood cell count (/µL)	10,200	3300–8600
Neutrophil percentage (%)	84.0	40.0–71.9
Lymphocyte percentage (%)	11.7	26.0–46.6
Aspartate aminotransferase (U/L)	19	13–30
Alanine aminotransferase (U/L)	21	7–30
γ‐Glutamyl transpeptidase (U/L)	62	9–32
Blood urea nitrogen (mg/dL)	13	8–20
Creatinine (mg/dL)	0.49	0.46–0.79
C‐reactive protein (mg/dL)	0.12	≦0.14
Creatine kinase (U/L)	177	41–153
Thyroid stimulating hormone (mIU/L)	1.040	0.610–4.230
Thyroxine (ng/dL)	1.73	0.93–1.70

**Figure 1:**
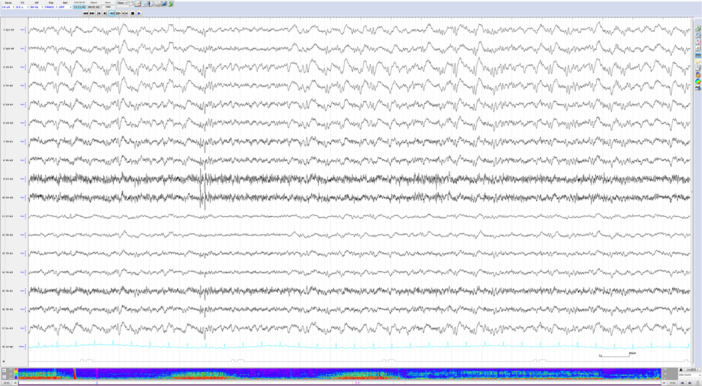
Electroencephalography on Day 2 of admission revealed low‐amplitude theta rhythms originating from the right frontal and right anterior temporal regions, which repeatedly evolved into rhythmic delta activity. Subsequently, generalized rhythmic spike‐and‐wave discharges at approximately 2 Hz were observed. Three episodes of epileptiform discharges were observed within the first 35 min of recording, prior to the intravenous administration of diazepam. The density‐modulated spectral array appeared to demonstrate repeated emergence and resolution of nonconvulsive status epilepticus. No epileptiform abnormalities were observed for 25 min following the administration of diazepam.

On Day 1 of hospitalization, 10 mg of intravenous diazepam was administered for the stupor, but no clinical improvement was observed. Her responsiveness fluctuated, ranging from no reaction at all to occasional scratching of her skin or muttering to herself, “Stay away from me!” Diurnal variations in autonomic symptoms, such as blood pressure and heart rate, as well as muscle tension, were also noted. EEG performed on Day 2 showed ∼5 Hz low‐amplitude theta rhythms originating from the right frontal and anterior temporal regions, which evolved into rhythmic generalized spike‐and‐wave discharges (Figure 1).

**Figure 2:**
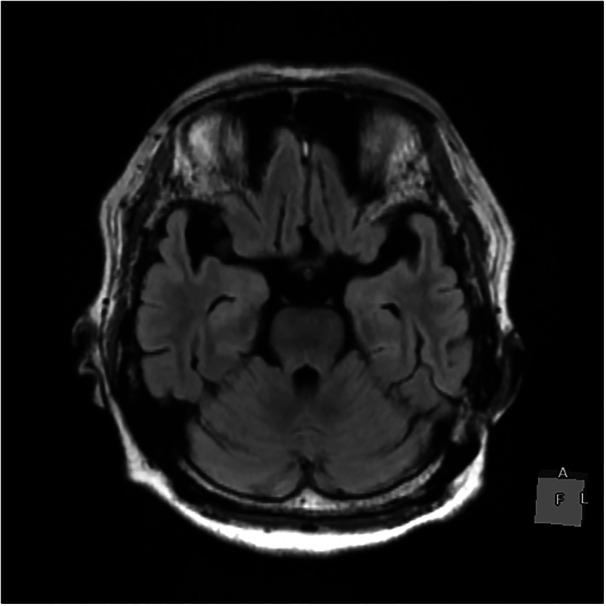
Brain magnetic resonance imaging revealed no lesions that could account for the catatonic state.

Intravenous administration of 10 mg diazepam eliminated the spike‐and‐wave discharges, but no improvement in her clinical condition was observed. Based on these findings, the patient was diagnosed with NCSE, and started on 2000 mg of levetiracetam. Suspecting encephalitis or encephalopathy, we performed a head magnetic resonance imaging (MRI) and cerebrospinal fluid analysis. The MRI showed no abnormalities (Figure 2), and cerebrospinal fluid analysis revealed a cell count of 1/μL, protein level of 31 mg/dL, glucose of 96 mg/dL, and negative oligoclonal bands. Additional tests for anti‐leucine‐rich glioma‐inactivated 1 protein antibody, anti‐contactin‐associated protein 2 antibody, and anti‐NMDA receptor antibody were also negative. The *Staphylococcus epidermidis* detected in the cerebrospinal fluid culture was determined to be a contaminant (Table 2).

**Table 2:** Results of the investigation into the causes of encephalitis and autoimmune disease.

Laboratory test	Results	Normal range
*Bacteriological investigation*		
Blood culture	Negative	
Cerebrospinal fluid culture	Methicillin‐resistant Staphylococcus epidermidis (1+)	
*Autoimmunological investigation*		
Serologic test for syphilis	Negative	
Interleukin 2 receptor (U/mL)	358	157–474
Anti‐nuclear antibody	40	<40
Anti‐SS‐A/Ro antibody	Negative	
Myeloperoxidase anti‐neutrophil cytoplasmic antibody (U/mL)	<1.0	<3.5
Anti‐thyroid peroxidase antibody	13	<16
Serum immunoglobulin level	Within normal limit	
Anti‐glutamic acid decarboxylase antibody	<5.0	<5.0
Anti‐GM1‐IgG antibody	Negative	
Antibody‐GQ1B IgG antibody	Negative	
Anti‐aquaporin 4 antibody	Negative	
Anti‐*N*‐methyl‐d‐aspartate receptor antibody	Negative	
*Cerebrospinal fluid investigation*		
Cell count	1	0–2
Protein	31	8–43
Glucose	96	50–75
Anti‐leucine‐rich glioma‐inactivated 1 protein antibody	Negative	
Anti‐contactin‐associated protein 2 antibody	Negative	

**Figure 3:**
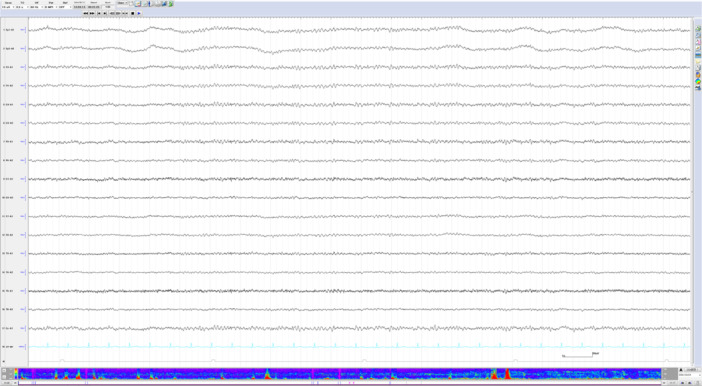
Electroencephalography on Day 7 of admission revealed no epileptiform discharges and showed a dominant 9‐Hz alpha rhythm.

On Day 7, EEG revealed a dominant 9‐Hz alpha rhythm (Figure 3). A continuous 49‐h EEG recording from Days 8 to 10 showed 8–10‐Hz alpha rhythms during wakefulness and normal sleep architecture, without epileptic discharges. Although EEG findings revealed no epileptic discharges, the stuporous state persisted. Consequently, we concluded that the patient had comorbid catatonia associated with schizophrenia and NCSE, and planned ECT. As the patient was unable to provide consent due to her stuporous state, informed consent was obtained from her brother as a surrogate decision‐maker. A total of six ECT sessions were performed, starting on hospital Day 21. Lorazepam 3 mg and levetiracetam 2000 mg were continued. On the day of ECT, the lorazepam dose was reduced to 2 mg by withholding 1 mg after breakfast. The levetiracetam dose was unchanged, but its administration time was shifted from after breakfast to after lunch. The first ECT session did not induce effective seizures, so the levetiracetam dose was reduced to 1000 mg, resulting in effective seizures. By the fourth session, the Bush–Francis Catatonia Rating Scale (BFCRS) score had decreased to 0, and ECT was completed after the sixth session. Following the resolution of the stuporous state, the patient reported, “I have no memory of being admitted. I felt like I was trapped in a terrifying world, hearing various voices, but I couldn't move or speak. Now, I don't hear those voices anymore, and my mind is clear.” Following the resolution of her stuporous state, the patient scored 29 out of 30 on the Mini‐Mental State Examination, indicating preserved global cognitive function. Neurological examination revealed no evidence of parkinsonism, motor weakness, cerebellar ataxia, or cranial nerve dysfunction. Moreover, *N*‐isopropyl‐p‐[¹²³I]iodoamphetamine single‐photon emission computed tomography demonstrated no abnormalities. These findings were not suggestive of an underlying neurodegenerative disorder.

On Day 45 of admission, the levetiracetam dose was tapered and discontinued. There was no recurrence of symptoms such as auditory hallucinations, delusions, or catatonia, nor were there any epileptic seizures. On Day 83, the patient was transferred back to A Hospital.

## DISCUSSION

This case involves a patient with catatonia associated with schizophrenia complicated by NCSE. The case met the diagnostic criteria for catatonia according to the DSM‐5. EEG repeatedly revealed low‐amplitude rhythmic theta waves that evolved in both space and time, eventually progressing to slow spike‐and‐wave complexes at approximately 2 Hz. Based on the Salzburg criteria,^10^ we diagnosed the patient with NCSE. However, despite improvement in the EEG findings with anti‐seizure medication, the catatonic state did not resolve. Consequently, ECT was performed, which led to the resolution of stupor, confirming the diagnosis of catatonic stupor associated with schizophrenia complicated by NCSE. To the best of our knowledge, only one previous report by Suzuki et al.^9^ has documented a similar case of catatonia associated with schizophrenia and NCSE. Unlike our case, their patient had a history of prior ECT treatment and exhibited motor seizures preceding NCSE.

Our EEG findings have two notable characteristics: (1) the findings are typical of NCSE, and (2) the NCSE resolved after 49 h of EEG monitoring following the administration of anti‐seizure medication.

### Catatonia in older patients

Late‐onset catatonia in older adults is often associated with underlying general medical conditions, including neurodegenerative disorders (e.g., dementia, Parkinson's disease), encephalitis, epilepsy, cerebrovascular disease, metabolic or infectious etiologies, endocrine dysfunction, nutritional deficiencies, or neoplastic processes.^11^ Catatonia may represent a maladaptive response to extreme fear or stress. In older adults, the emergence of catatonia may be attributed to the ageing process, the vulnerability provided by psychopathology, or emerging brain abnormalities.^12^

In the present case, no identifiable general medical condition was detected; therefore, the catatonic episode was diagnosed as a recurrence of the underlying schizophrenia spectrum disorder. Potential triggers for the catatonic episode, such as psychological stress or fear, were not clearly identified.

### Catatonia and epileptic seizures

The relationship between catatonia and epileptic seizures remains incompletely understood. Catatonia is believed to be associated with reduced γ‐aminobutyric acid (GABA) A activity.^13^ Suzuki et al. hypothesized that diminished GABA‐A activity could predispose patients with catatonic stupor to epileptic seizures.^9^ Both catatonia and NCSE can occur following benzodiazepine withdrawal and often improve with re‐administration of benzodiazepines.^14^, ^15^, ^16^ In our case, brain MRI, cerebrospinal fluid analysis, and antibody testing did not reveal any underlying encephalitis or encephalopathy that could have caused NCSE. On the day of admission, the patient was unable to take oral medication due to catatonia, which likely triggered benzodiazepine withdrawal and contributed to the onset of NCSE. The NCSE subsequently improved with the administration of benzodiazepines and anti‐seizure medication. However, since benzodiazepines were ineffective for treating catatonia in the context of schizophrenia, the patient ultimately achieved remission through ECT.

### ECT for NCSE and catatonia

Numerous reports have described catatonia caused by NCSE.^8^, ^17^ Ogyu et al. reported that the most common cause of NCSE was epileptic seizures in patients with epilepsy, while other causes included stroke, encephalitis, and medication withdrawal.^8^ They also noted that some patients had received methylprednisolone pulse therapy or hypothermia treatment, but none had undergone ECT. Suzuki et al. reported cases where electroconvulsive therapy was used for patients with catatonia associated with schizophrenia and co‐occurring NCSE.^9^ In their cases, NCSE followed an epileptic seizure related to catatonia in schizophrenia, and the stupor improved with ECT, even though anti‐seizure medication was ineffective. In contrast, our patient had no history of epilepsy but had a long‐standing history of schizophrenia. For over 30 years, the patient had remained stable without any auditory hallucinations or delusions. Unlike the previous case, our patient had no prior ECT treatment or history of tonic–clonic seizures. However, similar to the previous report, ECT was administered while the patient was on anti‐seizure medications.

### Epilepsy and ECT

While some reports have suggested that NCSE can occur after ECT,^18^ other studies have found that ECT does not induce epilepsy^19^ and can be safely performed in patients with epilepsy.^20^ Furthermore, ECT is effective in treating postictal catatonia.^21^ In our case, the patient received ECT while taking anti‐seizure medication. Although no effective seizures were elicited during the first session, increasing the stimulation intensity and reducing the dose of anti‐seizure medication resulted in successful seizures. No epileptic seizures occurred during the course of treatment, and ECT was safely administered. Although a history of epileptic seizures might make clinicians hesitant to use ECT, our findings suggest that ECT should still be considered in such cases.

## CONCLUSION

We encountered a case of catatonic stupor associated with schizophrenia and complicated by NCSE, which achieved remission following the administration of anti‐seizure medications and ECT. This case provides the following clinical insights: (1) stupor can be complicated by both NCSE and catatonia associated with schizophrenia; (2) when no physical cause of NCSE can be identified and clinical symptoms persist despite EEG improvement after antiepileptic drug therapy, the possibility of coexisting catatonia should be considered; and (3) ECT proved effective in treating catatonia associated with schizophrenia complicated by NCSE. Given the similarity in clinical symptoms between NCSE and catatonia, accurate differentiation is crucial, and awareness of their potential co‐occurrence is essential.

## AUTHOR CONTRIBUTIONS

Ayumi Takeshita interviewed and treated the patient, performed the literature search, and wrote and revised the first draft of the manuscript. Masaya Mashimoto interviewed and treated the patient and rewrote and revised the manuscript. Hiromi Chiba and Motohiro Ozone wrote and revised parts of the manuscript. All authors contributed to and approved the final version of the manuscript.

## CONFLICT OF INTEREST STATEMENT

The authors declare no conflicts of interest.

## ETHICS APPROVAL STATEMENT

This study was conducted in accordance with the principles outlined in the Declaration of Helsinki.

## PATIENT CONSENT STATEMENT

Written informed consent was obtained from the patient for presentation of their clinical course.

## CLINICAL TRIAL REGISTRATION

N/A.

## Data Availability

Research data are not shared.
